# The First Russian Seminar on Science Communication

**DOI:** 10.3325/cmj.2014.55.171

**Published:** 2014-04

**Authors:** Armen Yuri Gasparyan, Sergey V. Gorin, Alexander A. Voronov

**Affiliations:** 1 Chief Editor of *European Science Editing*; Council Member, European Association of Science Editors; Departments of Rheumatology and Research & Development, Dudley Group NHS Foundation Trust (Teaching Trust of University of Birmingham), Russells Hall Hospital, Dudley, United Kingdom; 2 Head of the Russian Regional Chapter of the European Association of Science Editors; Chief Editor of *International Scientific Researches*, Moscow, Russian Federation; 3 Department of Marketing and Trade Deals, Kuban State University, Krasnodar, Russian Federation

The standards of scholarly publications are evolving to meet the ever-growing demands of digital communications globally. Current authors, reviewers, editors, and publishers face numerous challenges, which can be overcome through Continuous Professional Development (CPD) aimed at advancing skills in research reporting and communicating with the global scientific community. The best scientific traditions nurtured by research and academic institutions in the Russian-speaking world over the past centuries may add to the quality of publications in the rest of the world. Conversely, the recent achievements in the systematization of scientific evidence and proper research reporting, made in Anglophone countries, are of great interest to the Russian counterpart, entering the global research competition.

**Figure 1:**
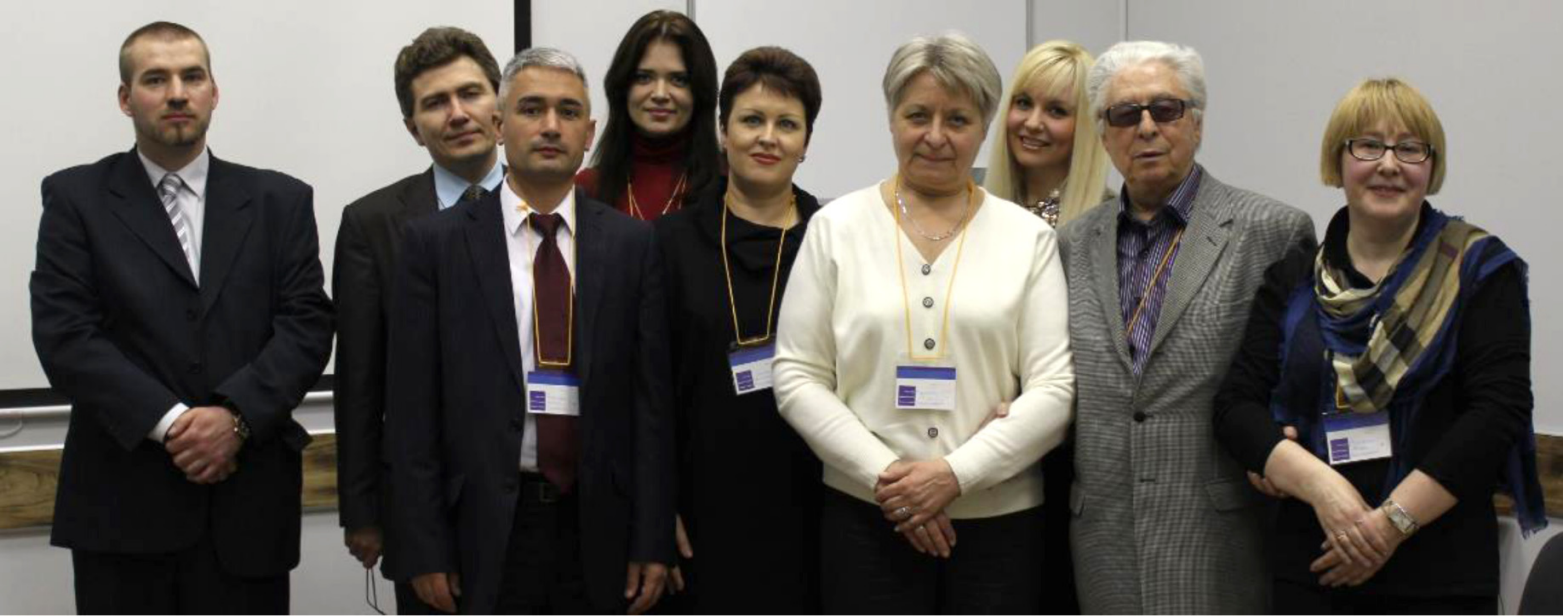
Members of the Russian Regional Chapter of the European Association of Science Editors at the First Russian Seminar on Science Communication (Moscow, March 25, 2014)

With that in mind, the Russian Regional Chapter of the European Association of Science Editors (EASE) organized its first seminar on science communication in Moscow on March 25, 2014 (Figure 1). It was chaired by Associate Professor, Dr Sergey V. Gorin and attended by a multidisciplinary group of researchers, journal editors, and experts in bibliographic databases from several leading Russian academic and research institutions in Moscow, Perm, Ryazan, and Yaroslavl. Dr Sergey Gorin opened the Seminar by presenting its aim and introducing lecturers. The aim was to improve research reporting and communication skills of Russian-speaking authors, reviewers, and editors and to facilitate their networking with collaborators from the English-language scientific journals and editorial associations such as EASE. The Russian chapter of EASE already arranged a regional meeting in 2013, published a paper in the *European Science Editing* journal, and translated the EASE guidelines for authors, which is now the main reference for most Russian-speaking authors from Russia and other European and Asian countries.

The first lecturer, Member of The Russian Academy of Natural Sciences, President of The League of Professional Image Makers, Prof Viktor M. Shepel reflected on philosophy of culture of scientific publications and shared his life-long experience of evaluating style and structure of multidisciplinary research publications. Prof Shepel emphasized the importance of novelty, creativity, and courteous style of research writing. Easily understandable and logical flow of ideas, correctly elaborated hypotheses, as well as brevity and evidence-based writing are all indicative of high culture of the authors. The authors who do not properly use scientific terms and excessively rely on professional slang distract the non-expert readership. Unfortunately, not all researchers are skilled to present details of methodology applicable to the concrete circumstances and aims of their research, which complicates the evaluation of the papers, particularly those written by PhD candidates. The authors should analyze and report whether the materials used for their studies are representative enough for the generalization of the results. Finally, each paper should have a limited, logically justified number of references to link each statement with a relevant scientific evidence or fact. Overly citing literature sources or quoting words of wisdom of eminent scientists do not allow the readership to judge the style of the authors. Generally, Russian-speaking researchers are modest in expressing their own style of writing and expressing ideas, and are too often critical toward their own research work.

The second talk was given by Prof Oleg S. Sukharev from the Institute of Economics of the Russian Academy of Sciences. He discussed the current standards of scholarly journal and book publishing, and criticized the widespread “obsession” with the numbers of papers at the expense of their quality. Monographs and other types of books are becoming increasingly unpopular among the authors because of the inappropriate crediting system and unnecessary prioritization of the long lists of publications and large citation numbers. Prof Sukharev reminded the audience of the long-forgotten principle “better fewer, but better.” He was also critical of ranking journals based solely on citation indicators and distinguishing the so-called elite journals. Many ground-breaking research papers are not highly cited despite having true impact on science and bringing prestige and even Nobel prizes to their authors. Moreover, not all citations in research papers are correct and relevant. Citation databases, and particularly the Russian Index of Scientific Citations (*http://elibrary.ru/*), still do not have strict criteria for calculating research performance indicators. This is why the interpretation of the citation counts and related indicators is often prone to bias. The lecturer also touched upon some rampant cases of plagiarism in Russian papers and its detection by reviewers and science editors. The plagiarism-detection software currently employed by journal editors calculates quantitative indicators that do not reflect the real picture of scientific misconduct. Plagiarism in research grant applications still goes unnoticed by most research evaluators. Some Russian scholarly journals lack traditions of peer review, which leads to a wide variability of its models across periodicals. Strangely, there are journal editors who consider “oral peer review” as viable as the written models.

The second session of the Seminar was run by Associate Professor, Dr Armen Yuri Gasparyan, who presented functional characteristics of bibliographic databases and search platforms, analyzed journal impact indicators, and introduced the EASE Statement on Inappropriate Use of Impact Factors (*http://www.ease.org.uk/publications/impact-factor-statement*) and the San Francisco Declaration of Research Assessment (*http://am.ascb.org/dora/*). He stressed the importance of opening access to scholarly publications, which may increase their influence on the global scientific progress. At the same time, Dr Gasparyan referred to the increasing number of retractions in English-language journals, which is largely due to their accessibility through the free full-text digital libraries and repositories. The subscription to periodicals and the distribution through the regular postal services are no longer capable of meeting the increasing demands of updated scientific information worldwide. Digitization, which includes the assignment of Digital Object Identifiers (DOIs), XML encoding for permanent archiving and preservation of scientific literature, indexing and posting full-texts of journal articles on digital platforms, is the requirement of the “big science” era. Many journals from non-Anglophone countries may improve their visibility and attractiveness for the global scientific community by following the best examples of online journals from Anglophone countries. That said, the authors should be also aware of the “predatory journals and publishers,” which are listed by Jeffrey Beall on his blog (*http://scholarlyoa.com*/). The blog contains information on unacceptable practices, which corrupt the open access and waste the authors’ efforts.

All the lectures were thoroughly analyzed and commented on by the Seminar participants at the roundtable discussion. The participants were encouraged by Dr Gasparyan to submit their best papers to the journals with wide visibility and international outreach. Some exemplary journals were discussed in detail (eg, *Croatian Medical Journal*). Prof Zhanna А. Мingaleva (Perm National Research Polytechnic Institute), Prof Svetlana A. Gusar (Moscow State University of Economics, Statistics and Informatics), Olga V. Kirillova (NEICON School in Moscow), and Elena V. Karpunina (Intereconom Publishing) shared their thoughts on the visibility of Russian journals in international bibliographic databases and reassured that most regional journals are now correcting format of references and upgrading standards of peer review and ethical publishing, which will be helpful for indexing in the future.

The Seminar participants were awarded with certificates credited with CPD credits in research methodology and communication. It was decided to arrange such seminars with international participation annually to discuss matters arising across multidisciplinary journals and to advance science communication skills of non-native English-speaking authors, reviewers, and editors of regional journals.

